# Establishing efficient multi-gene editing tools for papaya

**DOI:** 10.1093/hr/uhag049

**Published:** 2026-02-20

**Authors:** Bowei Wang, Xuesong Cao, Zeng Lin, Yiting Zhuang, Guihua Yang, Jian-Kang Zhu, Ray Ming, Jingjing Yue

**Affiliations:** Center for Genomics and Biotechnology, Fujian Provincial Key Laboratory of Haixia Applied Plant Systems Biology, Key Laboratory of Genetics, Breeding and Multiple Utilization of Crops, Ministry of Education, Fujian Agriculture and Forestry University, Fuzhou 350002, Fujian, China; Institute of Advanced Biotechnology and School of Life Sciences, Southern University of Science and Technology, Shenzhen 518055, China; State Key Laboratory of Tropical Crop Breeding, Institute of Tropical Bioscience and Biotechnology, Sanya Research Institute, Chinese Academy of Tropical Agricultural Sciences, Sanya, Hainan 572000, China; Center for Genomics and Biotechnology, Fujian Provincial Key Laboratory of Haixia Applied Plant Systems Biology, Key Laboratory of Genetics, Breeding and Multiple Utilization of Crops, Ministry of Education, Fujian Agriculture and Forestry University, Fuzhou 350002, Fujian, China; Center for Genomics and Biotechnology, Fujian Provincial Key Laboratory of Haixia Applied Plant Systems Biology, Key Laboratory of Genetics, Breeding and Multiple Utilization of Crops, Ministry of Education, Fujian Agriculture and Forestry University, Fuzhou 350002, Fujian, China; College of Life Sciences, Fujian Agriculture and Forestry University, Fuzhou 350002, Fujian, China; Center for Genomics and Biotechnology, Fujian Provincial Key Laboratory of Haixia Applied Plant Systems Biology, Key Laboratory of Genetics, Breeding and Multiple Utilization of Crops, Ministry of Education, Fujian Agriculture and Forestry University, Fuzhou 350002, Fujian, China; College of Life Sciences, Fujian Agriculture and Forestry University, Fuzhou 350002, Fujian, China; Institute of Advanced Biotechnology and School of Life Sciences, Southern University of Science and Technology, Shenzhen 518055, China; Center for Genomics and Biotechnology, Fujian Provincial Key Laboratory of Haixia Applied Plant Systems Biology, Key Laboratory of Genetics, Breeding and Multiple Utilization of Crops, Ministry of Education, Fujian Agriculture and Forestry University, Fuzhou 350002, Fujian, China; Department of Plant Biology, University of Illinois at Urbana-Champaign, Urbana, IL 61801, USA; Center for Genomics and Biotechnology, Fujian Provincial Key Laboratory of Haixia Applied Plant Systems Biology, Key Laboratory of Genetics, Breeding and Multiple Utilization of Crops, Ministry of Education, Fujian Agriculture and Forestry University, Fuzhou 350002, Fujian, China; College of Horticulture, Fujian Agriculture and Forestry University, Fuzhou 350002, Fujian, China

## Abstract

Papaya is a major tropical fruit crop with notable nutritional and economic value, yet its genetic improvement through modern breeding technologies faces substantial challenges. The traditional tissue culture process is both labor-intensive and time-consuming, causing gene-editing advancements in papaya to lag behind those in other crops. To overcome these obstacles, we developed a tissue culture–independent hairy root system in papaya, which enables efficient gene editing and significantly enhances the application and development of editing tools. This innovative platform allows for the pre-assessment of editing efficiency and supports the establishment of adenine base editor (ABE) and cytosine base editor (CBE) tools in papaya, thereby mitigating the high failure costs associated with the lengthy cycle of conventional genetic transformation. Utilizing this system, we pre-tested sgRNA activity and achieved high editing efficiency of *CpWIP3* during stable transformation. Additionally, through promoter screening, we successfully developed ABE and CBE tools, marking the first precise single-nucleotide editing system in papaya. This gene-editing system provides a crucial platform for advancing functional genomics and accelerating precision breeding in papaya.

## Introduction

Papaya (*Carica papaya* L.) is one of the world's top 10 tropical fruits, belonging to the family Caricaceae, which comprises six genera and 40 species [1]. The ripe papaya fruit is rich in vitamins A, B, and C, as well as minerals, such as potassium, magnesium, and boron, along with dietary fiber [1]. Papaya is exceptionally high in vitamin A, making it a recommended dietary intervention to prevent vitamin A deficiency in developing countries. Rich in papain, papaya holds significant economic value due to its extensive applications in the pharmaceutical, food, and cosmetic industries [2]. As a globally important tropical fruit crop, papaya ranks third in production worldwide, contributing to 15.36% of total tropical fruit output [3]. However, papaya production still faces numerous challenges, including devastating pests and diseases (such as yield losses caused by papaya ringspot virus [PRSV]) and low genetic diversity and sex separation [3]. Recent advances in biotechnological breeding now offer new opportunities for papaya improvement. Modern techniques, such as transgene and genome editing, enable more precise cultivar improvement. Notably, researchers have successfully used CRISPR/Cas9 to edit the papaya genome [4], demonstrating the potential of precise genetic improvement in this crop. Despite the emergence of genome editing technologies in the papaya species, their application has been relatively slow. To accelerate the use of genome editing in papaya, it is crucial to continuously optimize the papaya genetic transformation system, shorten transformation time, and urgently continue the development of efficient and precise genome editing systems.

Genome editing, particularly the CRISPR/Cas9 system, has become a significant breakthrough in biotechnology recently. This method uses a specific RNA to direct the Cas9 enzyme to create breaks in the plant's DNA. The cell then repairs these breaks using its natural repair mechanisms, which can sometimes introduce undesired insertions or deletions (indels), thereby affecting the precise modification [5]. To overcome these issues, researchers have developed CRISPR/Cas9-mediated base-editing technology. Two classes of DNA base editors have been described to date: cytosine base editors (CBEs) and adenine base editors (ABE), which can precisely induce single-base changes, such as converting an adenine (A) to a guanine (G) or a cytosine (C) to a thymine (T) [6, 7]. Base editing (BE) has enabled precise trait improvements in key crops. For example, cytosine BE of the acetolactate synthase (ALS) gene in rice (*OsALS1* P171 → P171F) has conferred broad-spectrum herbicide tolerance [8]. In tomato, multiplex Target-AID BE of carotenoid-biosynthesis regulators (*SlDDB1*, *SlDET1*, *SlCYC-B*) has resulted in fruit lines with elevated lycopene and β-carotene [9]. These representative cases illustrate the application of BE in engineering herbicide resistance, nutritional enhancement, and even flowering time (developmental) traits in crops.

However, the papaya transformation system still faces numerous limitations that hinder its biotechnology development and application, and slow the progress of precision breeding. In papaya, traditional *Agrobacterium*-mediated transformation is characterized by low efficiency, extended transformation cycles, and a strong influence from varietal differences, limiting its use in functional genomics and breeding [10, 11]. These factors ultimately result in longer breeding cycles and less stable outcomes. A novel method called ‘cut–dip–budding’ (CDB) has recently been developed that eliminates the need for tissue culture in transformation. In the CDB technique, various explants are able to dip in an *Agrobacterium* suspension, which induces transgenic hairy roots from which shoots can subsequently bud and grow into plantlets [12]. The simplified CDB system enables rapid validation of gene-editing tools in hairy roots and achieves stable transformation by exploiting root regeneration. It has been successfully applied in dandelion, sweet potato, woody plants, and succulents, providing a simple and versatile approach for genetic improvement of recalcitrant species [12].

In this study, we pioneered a simplified hairy root transformation method that significantly improves early screening for high-efficiency CRISPR/Cas9 editing sites. Using this approach, we developed ABE and CBE systems specifically for papaya. This study showcases an advanced genetic transformation platform for papaya, capable of supporting both CRISPR/Cas9-mediated gene-editing and base-editing applications. It is anticipated that this platform will play a crucial role in facilitating the application of precise genome-editing technologies for the genetic enhancement of papaya.

## Results

### Establishment of a streamlined system for inducing hairy roots in papaya

**Figure 1:**
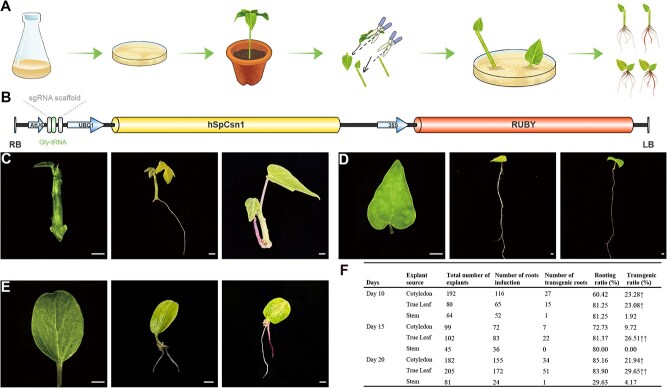
CDB delivery system in *Carica papaya* L. (A) Workflow of the CDB delivery system. *A. rhizogenes* K599 carrying the RUBY marker was inoculated onto 10- to 20-day-old papaya seedlings. Constructs were delivered into cells at cut sites, inducing hairy roots within 4 weeks. RUBY-positive roots were identified by reporter expression. (B) Schematic representation of the RUBY reporter gene vector. (C–E) Induction of RUBY-positive hairy roots from stem segments, true leaves, and cotyledonary explants. (F) Quantification of total hairy roots and RUBY-positive hairy roots obtained from explants of 10-, 15-, and 20-day-old seedlings inoculated with *A. rhizogenes* K599. Arrows indicate descriptive increases. Scale bars, 1 mm.

The CDB delivery system enables genetic modification without the need for complex tissue culture procedures [12]. However, conventional papaya genetic transformation remains constrained by lengthy tissue culture cycles, which hampers the application of genome editing tools and genetic breeding [13]. We thus set out to develop a tool that supports the establishment of a simplified transformation system for papaya, to advance genome editing tool development and expedite gene function verification. In our approach, we employed the RUBY reporter gene as a visual marker, enabling the identification of transformed roots without chemical treatments or specialized equipment [14] (Fig. 1A). Additionally, we employed *Agrobacterium rhizogenes* K599 to assess the efficiency of hairy root induction across various papaya explants (Fig. 1A and B).

Papaya seedlings aged 10, 15, and 20 days, along with various papaya explants including stem segments, true leaves, and cotyledons, were excised and inoculated with *A. rhizogenes* K599 at the incision site. These were subsequently cultured in vermiculite within culture boxes, adhering to the protocol of CDB (Supplementary Figs. S1 and S2). After 30 days, the papaya explants developed hairy roots, some of which were RUBY-positive roots (Fig. 1C–E).

The proportion of transgenic positive roots originating from papaya stems was the lowest when compared to cotyledons and true leaves, with rates of 1.92%, 0%, and 4.17% in seedlings aged 10, 15, and 20 days, respectively (Fig. 1F). Analyzing the transformation efficiency of true leaves and cotyledons across three developmental stages, true leaves exhibit transformation rates of 23.08%, 26.51%, and 29.65%, compared to cotyledons, which showed rates of 23.28%, 9.72%, and 21.94% in 10-day, 15-day, and 20-day seedlings, respectively (Fig. 1F). Although no biological replicates were available for statistical testing, numerical differences in transformation ratios were still observed among different explant types and across different time points. This suggests that true leaves of 10-day-old seedlings were the most suitable explants for conducting hairy root induction.

### Hairy root systems enable early prediction of editing efficiency in papaya

We utilized the papaya hairy root platform as a tool to accelerate CRISPR/Cas9 validation before proceeding with stable transformations. Papaya serves as an ideal model for these studies due to its distinctive sex determination mechanism [15]. We selected two candidate genes associated with sex differentiation and assessed their targeted mutagenesis using the hairy root system.

One of the genes, *CpGASA*, includes a pair of homologous genes on the sex chromosomes (*CpGASA_X*, *CpGASA_HSY*), which are homologous to the *GASA* gene in Arabidopsis (Supplementary Figs S3A–B and S4A; Table S1)**.** The *GASA* gene belongs to a class of gibberellin (GA)-induced genes encoding small cysteine-rich peptides that primarily regulate and participate in the normal development of floral organs [16]. The other gene, *CpWIP3*, is a homolog of the NAC transcription factor family. In melon, the homologous gene *CmWIP1* is highly expressed in the male flowers, where it suppresses pistil development by repressing *CmACS7* [17] (Supplementary Figs S3A, C, and  S4B; Table S1). We employed the papaya hairy root system to assess the knock-out efficiency of these two candidate male-promoting genes with four sgRNAs, as well as their double knockout efficiency, to preliminarily verify the effectiveness of gene editing (Supplementary Table S2)**.**

**Figure 2:**
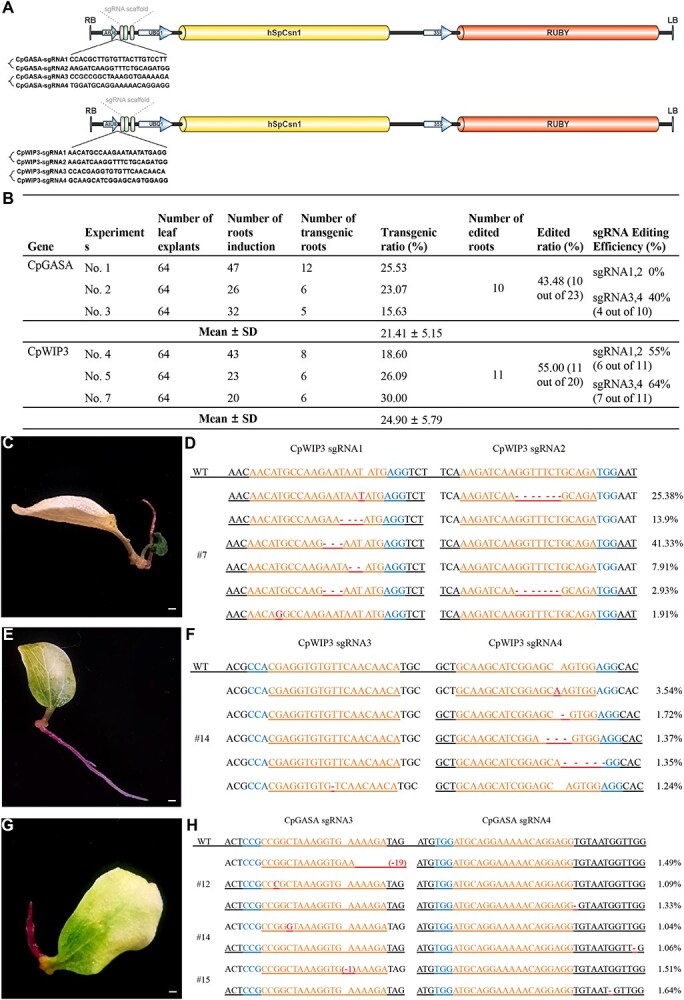
Gene editing of *CpGASA* and *CpWIP3* genes using the CDB delivery system. (A) Schematic representation of the *CpGASA* and *CpWIP3* gene-editing vectors. (B) Efficient delivery of gene-editing constructs via the CDB system, generating edited hairy roots in three independent experiments. Values represent mean ± SD from three independent experiments (n = 3). Different genes were compared using Student's *t*-test (*P* > 0.05). (C, E) Induction of transformed hairy roots and regenerated shoots from explants inoculated with the *CpWIP3* gene-editing vector. (D, F) Sequencing analysis of *CpWIP3* target sites. (G) Induction of transformed hairy roots from explants inoculated with the *CpGASA* gene-editing vector. (H) Sequencing analysis of *CpGASA* target sites. The sgRNA target sequence, PAM region, and mutations are indicated accordingly in the sequence alignment. WT, wild type. Scale bars, 1 mm.

We constructed expression cassettes with AtU6-driven sgRNAs and AtUBQ1-driven Cas9, positioned before the RUBY cassette, enabling the simultaneous delivery of sgRNAs, Cas9, and the RUBY reporter into plant cells (Fig. 2A). We designed two sgRNAs for each gene, *CpGASA* and *CpWIP3,* using tRNA^Gly^ as a linker to target the exonic regions for these two genes. The four vectors, pAtU6::CpGASA–sgRNA1–sgRNA2, pAtU6::CpGASA–sgRNA3–sgRNA4, pAtU6::CpWIP3–sgRNA1–sgRNA2, and pAtU6::CpWIP3–sgRNA3–sgRNA4, were introduced into the petiole sites of papaya cotyledons via *A. rhizogenes* K599-mediated transformation (Supplementary Table S3). In three independent hairy-root transformation experiments targeting the papaya *CpGASA* and *CpWIP3* genes, a high proportion of the regenerated transgenic roots carried CRISPR/Cas9-induced edits at the target loci. High-throughput amplicon sequencing of these loci revealed that approximately 43% of the transgene-positive hairy roots were edited at all intended target sites. Similarly, around 55% of the transgenic roots obtained for *CpWIP3* contained edits at all target sites (Fig. 2B). Transgenic ratios are presented as mean ± SD from three independent experiments (*n* = 3). Differences between *CpGASA* and *CpWIP3* were assessed using Student's *t*-test and were not statistically significant (*P* > 0.05). Among the sgRNA pairs tested, the *CpWIP3* sgRNA3,4 and *CpGASA* sgRNA3,4 yielded particularly high editing frequencies, whereas the *CpGASA* sgRNA1,2 pair did not produce detectable mutations (Fig. 2B; Supplementary Fig. S5A and B; Tables S4 and S5).

Amplicon sequencing revealed a variety of mutation types, primarily small insertions and deletions, with occasional single-nucleotide substitutions occurring at the Cas9 cut sites (Fig. 2C–H; Supplementary Fig. S6A–C). Notably, most edited samples exhibited mutations at only one of the two target loci, rather than simultaneous edits at both sites. Collectively, these findings demonstrate the efficacy of the hairy-root system as a robust platform for rapid, preliminary testing of CRISPR/Cas9 editing constructs in papaya.

### Pre-validation using hairy roots enhances the efficiency of CRISPR/Cas9 editing in the stable transformation of papaya

**Figure 3:**
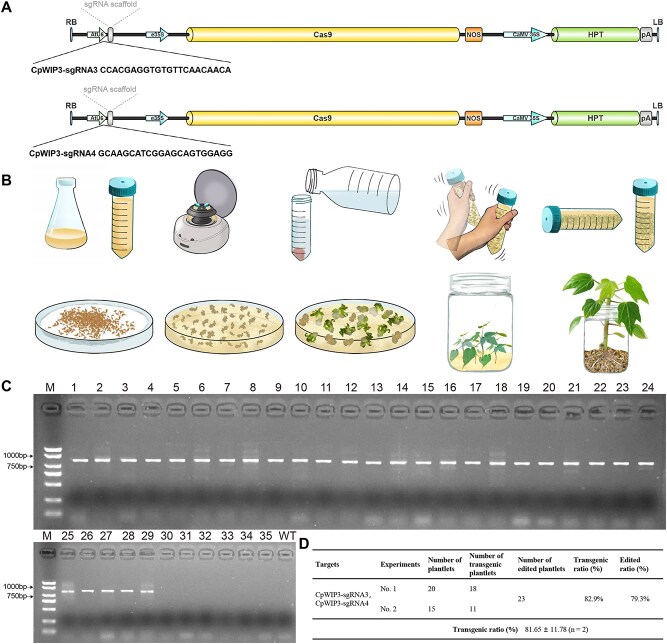
*A. tumefaciens*-mediated transformation of papaya embryogenic callus. (A) Schematic diagram of the *A. tumefaciens*-mediated transformation system. (B) Schematic representation of stable transformation using an sgRNA expression cassette. (C) PCR analysis of putative transgenic papaya T_0_ explants with gene-specific primers targeting the *CpWIP3* knockout construct. (D) Statistics of transgenic and edited plantlets generated through the stable and efficient genetic transformation system.

After utilizing the hairy root method to screen for CRISPR/Cas9 *CpWIP3* sgRNA3,4, we process stable transformation to produce edited plants. To facilitate this process, we have optimized the genetic transformation system for papaya. Initially, we employed the effective three-step callus induction protocol from our previous study [18]. However, we improved upon this by avoiding the use of hypocotyls as explants, which showed a contamination rate of up to 60%. Instead, we utilized embryos from mature seeds for callus induction, resulting in nearly contamination-free cultures and markedly higher induction rates and callus activity (Supplementary Fig. S7A). Obtaining high-quality embryogenic callus is the first step towards successful transformation. We selected the CpWIP3–sgRNA3 and CpWIP3–sgRNA4 targets, which were identified as optimal target sgRNAs for stable transformation (Fig. 3A). We explored the optimal transformation conditions and found that infecting the embryogenic callus with *Agrobacterium* at an OD value of 0.8 for 25 min, followed by a 5-min shaking, yielded the best results. Additionally, by experimenting with the differentiation medium, we determined that the optimal hormone ratio in the differentiation medium is 0.2 mg/L of 6-BA and 0.2 mg/L of NAA, which markedly improved shoot regeneration efficiency (Fig. 3B).

Finally, 35 independent papaya regenerants were screened, and 29 lines were transgenic positive plants, demonstrating the effectiveness of the transformation and editing methods (Fig. 3C and D; Supplementary Table S6). In total, we detected CRISPR/Cas9-induced mutations at the *CpWIP3* locus in 23 of 29 transgenic lines, achieving a 79.3% efficiency (Fig. 3D). Among these 23 mutants, 21 exhibited homozygous edits at the CpWIP3–sgRNA3 target site, while 2 carried homozygous edits at the CpWIP3–sgRNA4 site. Sequence analysis revealed that single-nucleotide base substitutions were the predominant mutation type (Supplementary Table S6). Notably, both sgRNAs were pre-validated using a hairy-root assay before transformation to ensure they were highly active guides. This in vivo screening process resulted in exceptionally high editing rates.

### Establishing a high-efficiency ABE8e base-editing system in papaya

**Figure 4:**
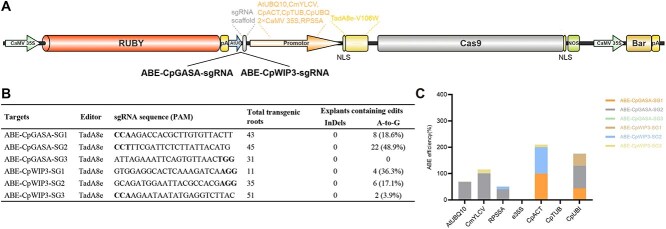
Development of ABE in papaya. (A) Schematic diagrams of the ABE constructs used in this study. (B) Genotyping results of explants edited with ABE (A to G edit efficiency: The ratio of A to G edits to the total number of transgenic roots). (C) Editing efficiencies at the *CpGASA* and *CpWIP3* loci in transgenic explants, with ABE driven by the indicated promoters (ABE efficiency: The ratio of A to G edits to the number of transgenic roots sequenced).

In current plant research, CRISPR/Cas9 systems predominantly induce small insertions or deletions, with precise single-nucleotide substitutions being a rarity [19]. To enable efficient BE in papaya, we developed an ABE8e-based system and systematically compared seven constitutive promoters: four commonly used heterologous promoters (*AtUBQ10*, *CmYLCV*, *RPS5A*, and *2 × CaMV 35S*) and three endogenous papaya promoters (*CpACT*, *CpTUB*, and *CpUBQ*) [20], which were selected from genes with high expression levels (FPKM >50) (Fig. 4A; Supplementary Table S7 and S9).

The editing outcomes varied markedly with promoter choice. The sequencing of transgenic hairy roots showed that editing occurred at the ABE–CpGASA–SG2 locus, where the pCmYLCV-driven ABE8e achieved highly efficient A-to-G conversion (100% efficiency, the ratio of 10 edited roots to the 10 transgenic roots sequenced). Similarly, pCpACT drove highly efficient editing at both the ABE–CpGASA–SG1 and ABE–CpWIP3–SG2 targets (100% editing, the ratio of four edited roots to the four transgenic roots sequenced) (Fig. 4B and C; Supplementary Tables S11 and S12; Fig. S8B–F). These results clearly demonstrate the high activity of the CmYLCV promoter, consistent with reports that its expression level is comparable to or even stronger than that of the *2 × CaMV 35S* ‘super-promoter’ [21].

Overall, our findings highlight the importance of promoter selection in determining base-editing efficiency in papaya. By utilizing the strong pCpACT and pCmYLCV promoters, we have developed an effective ABE8e platform for precise single-nucleotide substitutions in papaya, demonstrating its potential utility in this challenging fruit crop. The use of the hairy root system further facilitates the selection of multiple promoters, underscoring their critical role in enhancing base-editing efficiency.

### Programmable CBE single-nucleotide editing using CDB delivery-optimized base editors

**Figure 5:**
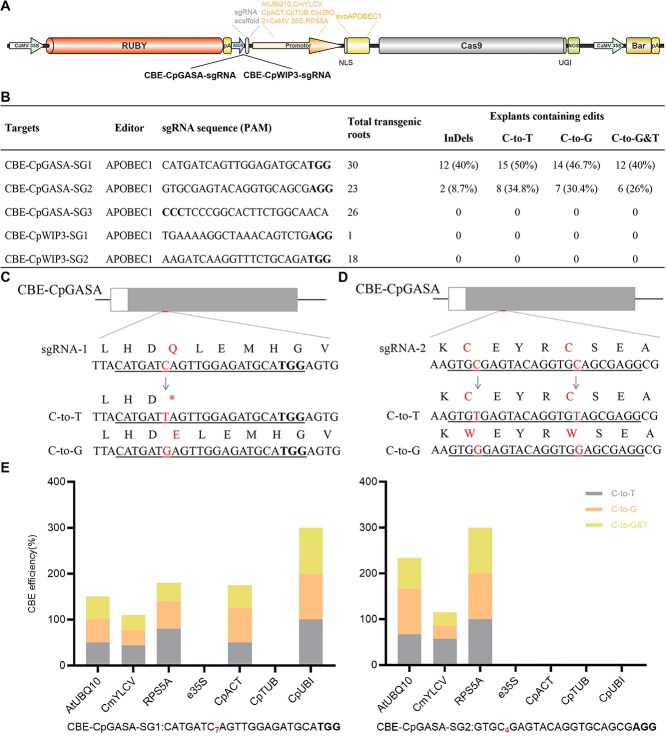
Development of CBEs in papaya. (A) Schematic diagrams of the CBE constructs used in this study. (B) Genotyping results of explants edited with CBE (edit efficiency: The ratio of edits to the total number of transgenic roots). (C, D) Schematic illustrations of the target sites within the *CpGASA* gene and the expected editing outcomes mediated by CBE, stop codons are indicated by an asterisk (*). (E) Editing efficiencies at the *CpGASA* locus in T_0_ transgenic explants, with CBEs driven by the indicated promoters (edit efficiency: The ratio of edits to the number of transgenic roots sequenced).

To establish cytosine BE in papaya, we adapted an evolved Rattus norvegicus APOBEC1 deaminase (evoAPOBEC1) to construct a CGBE system [22]. This platform combines Cas9 nickase (Cas9n) with evoAPOBEC1 and the uracil glycosylase inhibitor (UGI) [23]. A RUBY reporter was employed to facilitate the hairy root system (Fig. 5A; Supplementary Fig. S8A).

The choice of promoter was crucial for efficient editing. Utilizing the papaya ubiquitin promoter (pCpUBI), we achieved C-to-base conversions at the CBE–CpGASA–SG1 locus with 60.0% (the ratio of 18 edited roots to the 30 total transgenic roots) efficiency (Fig. 5B; Supplementary Tables S13 and S14). These conversions included canonical C-to-T edits, introducing a premature stop codon (CAA/CAG → TAA/TAG), as well as unexpected C-to-G transversions that converted glutamine (Q) to glutamic acid (E) (Fig. 5C; Supplementary Fig. S9). At the CBE–CpGASA–SG2 site, the ribosomal protein promoter pRPS5A exhibited even stronger activity, with certain events demonstrating complete (100%, the ratio of two edited roots to the two transgenic roots sequenced) C-to-G and C-to-T conversion at the target cytosine (Fig. 5D and E; Supplementary Fig. S9; Tables S13 and S14). This promoter-dependent variation is consistent with previous findings that pRPS5A enhances base-editing efficiency in plants [24].

In contrast, no editing activity was detected at CBE–CpGASA–SG3 or the tested *CpWIP3* locus under any promoter configuration (Supplementary Tables S13 and S14). A plausible explanation is that local chromatin accessibility restricts Cas9–deaminase binding and activity [25]. These results demonstrate that optimized promoters such as pCpUBI and pRPS5A enable very efficient cytosine editing at certain papaya loci, although editing efficiency varied among different targets.

## Discussion

However, the implementation of CRISPR/Cas9 in papaya breeding remains limited, primarily due to the challenges associated with genetic transformation [11]. Since its initial application to plant genome editing in 2013, CRISPR/Cas9 technology has been rapidly embraced by researchers, significantly advancing the field of plant genetics [26]. To address this, we utilized a rapid and efficient papaya hairy root system via the CDB delivery method, which has provided an effective platform for the development and validation of gene-editing tools (Fig. 1A). This system allowed us to prevalidate the efficiency of gene-editing targets, thereby accelerating experimental workflows. Leveraging this platform, we developed, for the first time, both ABE and CBE gene-editing tools in papaya.

Previous studies have improved plant-based editors by incorporating evolved deaminases. Recent refinements using evolved deaminases have greatly enhanced BE efficiencies: for example, a TadA8e–V106W ABE (with sgRNAs under the rice U6 promoter and Cas9n under the maize ubiquitin promoter) markedly increased A to G editing at NGG and even NG PAM sites [27, 28]. Similarly, CBEs constructed with an evolved cytidine deaminase (evoAPOBEC1 fused to UGI) significantly improved on-target C to T editing in rice and tomato [29]. By enabling direct base conversions without creating DSBs, BE offers high-precision editing with minimal indels compared to standard Cas9 nuclease approaches [30]. However, base editors remain constrained by narrow editing windows and strict PAM requirements, and their performance is highly dependent on promoter strength [24]. The notoriously low transformation efficiency in papaya has further hindered the deployment of BE tools in this species [31]. To overcome this limitation, we developed papaya ABE and CBE toolboxes utilizing a hairy root system, allowing for direct comparison of the efficiencies of different BE constructs and sgRNAs. The ABE system integrated TadA8e–V106W fused to Cas9n under various promoters, while the CBE system employed evoAPOBEC1–Cas9n–UGI fusions driven by heterologous promoters (Supplementary Fig. S8A). This setup enabled a systematic comparison of seven promoters and 11 NGG-PAM sgRNA targets in hairy roots (Supplementary Tables S7 and S8). High editing efficiencies were observed across several promoter–target combinations: (i) pCpUBI-driven ABE at CpGASA–SG2, (ii) pCpACT-driven ABE at CpWIP3–SG2, and (iii) pCpUBI-driven CBE at CpGASA–SG1 (Figs 4C and 5E). Our hairy-root platform offers a practical framework for optimizing BE components, such as promoters, editors, and guide RNAs, within plant systems. Editing efficiency is influenced by the promoter strength and expression profile, the spatiotemporal expression of the Cas9 editor during regeneration, and the coupling between target site characteristics and editor type. Viral promoters like pCmYLCV drive high expression in rapidly dividing cells, while more moderate endogenous promoters like pCpACT can improve editing efficiency by reducing selection bias against heavily edited cells [24]. These differences reflect promoter–context interactions, rather than inherent limitations of specific promoters. These results indicate that editing efficiency is strongly influenced by promoter choice, as well as the context of the target site, aligning with previous research [32].

However, we acknowledge that existing BE technologies face inherent limitations, including transition-only edits and PAM dependency, highlighting the necessity for ongoing optimization [30]. In our system, despite the inclusion of UGI to inhibit UNG, we still observed C-to-G conversions, likely due to residual activity of the base excision repair (BER) pathway. When APOBEC1 deaminates cytosine to uracil, incomplete UNG inhibition may allow uracil excision and formation of an abasic (AP) site, which can be repaired by inserting a guanine, resulting in unintended C-to-G edits [33]. Although infrequent, such events can give rise to chimeric or individual mutant plants during regeneration, which require sequencing-based screening to evaluate their potential impact on editing precision.

CRISPR/Cas9 genome editing has created new opportunities for targeted trait improvement, yet practical application is often constrained by tissue culture–dependent transformation systems. Traditional methods, such as *Agrobacterium*- or biolistic-mediated transformation, involve prolonged culture steps, plant regeneration, and typically result in low editing efficiency. In contrast, the CDB-based hairy root transformation system facilitates rapid sgRNAs screening and editing assessment, yielding results within a month. This approach circumvents the need for aseptic culture conditions, allowing for efficient evaluation of sgRNAs activity prior to committing to stable transformation. In this study, we designed editing vectors targeting *CpGASA* and *CpWIP3*. Using the CDB hairy root system, we achieved editing efficiencies of ~50% across transformed hairy roots (Fig. 2B). The prescreening step significantly boosted the success rate of papaya editing. In contrast, earlier attempts at papaya transformation using unvalidated guides achieved mutation frequencies of only 0.15% to 3% [11], highlighting the substantial improvement in efficiency provided by the hairy-root prescreening. The stable genetic transformation system we developed represents a significant improvement over previous transformation processes [34, 35]. From seed embryos to fully regenerated transgenic plants, the process takes only 8 months, which is considerably shorter than the previously reported 12 to 15 months, greatly enhancing research and breeding efficiency.

In tissue culture–derived transgenic papaya seedlings, CRISPR/Cas9 editing efficiencies reached 82.9% at CpWIP3–sgRNA3 and CpWIP3–sgRNA4 (Fig. 3D). However, editing outcomes at these sites were relatively homogeneous, necessitating extensive genotyping in regenerated seedlings for validation. It is important to note that we cannot directly use the CDB method to obtain gene-edited plants because we have not yet successfully achieved the step of regenerating shoots from edited roots. This challenge may stem from the significantly varying capacities for root-to-shoot regeneration across different species, compounded by the low degree of tissue differentiation, suboptimal culture conditions, and the limitations imposed by the genetic background of papaya [18, 36]. Therefore, once we accomplish the root-to-shoot regeneration process in the future, we will no longer need to rely on tissue culture to obtain gene-edited plants, significantly reducing the time required.

As gene-editing technology rapidly develops, we can now achieve targeted genetic improvements in plants in ways that were previously unattainable with traditional methods [37]. By integrating genomic information with precise gene editing into traditional papaya breeding, we can rapidly and accurately improve papaya traits such as sex determination and fruit quality [38, 39]. Through precise editing of sex-determining genes, we can produce transgenic papaya plants with non-segregating sex traits.

**Figure 6:**
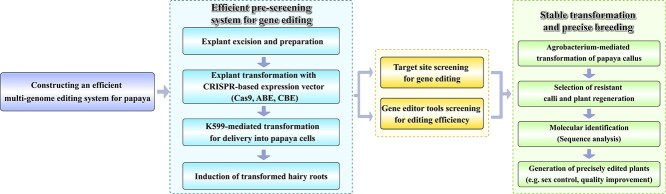
Development of an efficient pre-screening and stable genetic transformation system for papaya breeding.

To apply these advancements to papaya breeding, we developed a high-precision gene-editing platform designed to establish a precision papaya breeding system (Fig. 6), which comprises three key modules. Looking ahead, we plan to identify candidate genes through genome-wide association studies and leverage this platform to integrate genome-editing breeding with conventional hybrid breeding. The establishment of this platform not only enhances the efficiency and precision of papaya genetic improvement but also provides robust support for future papaya breeding efforts.

## Materials and methods

### Plant material and growing conditions

Seeds of the papaya *Zhongbai* [18] variety were surface-sterilized by soaking in 0.2% (2 g/L) metribuzin solution for 30 min, rinsed with distilled water, and then soaked in 100 mg/L gibberellin (GA_3_) for 1 h. After another rinse, the seeds were soaked in 1% (10 g/L) sodium bicarbonate (NaHCO_3_) solution for 8 h. Sterilized seeds were then incubated on moist filter paper in Petri dishes at 28 ± 2°C, with water added every 2 days to maintain moisture. When the seeds turned white, they were sown into a substrate (nutrient soil: vermiculite, 3:1). The substrate was treated with imidacloprid (water: imidacloprid = 5000:2) and oxacillin (1000:1) to prevent pests and diseases, and the seedbed temperature was maintained at 28 ± 2°C. Papaya seedlings were successfully obtained.

### Gene cloning and dual-site knockout vector construction

A CRISPR/Cas9 vector containing the RUBY reporter gene was constructed with two independent U6-driven sgRNA cassettes for multiplex genome editing [40]. Target sequences were designed using the CRISPR-P 2.0 tool [41], and four sgRNAs targeting the *CpGASA* and *CpWIP3* genes were selected, with sgRNA1 and sgRNA2 assembled into one construct and sgRNA3 and sgRNA4 into a second. For dual-site targeting, primers encoding 20-bp target sequences were synthesized and used to PCR-amplify Gly–tRNA–sgRNA scaffold fragments (PrimeSTAR® Max DNA Polymerase, TaKaRa, Japan). These fragments were cloned into the CRISPR vector via Golden Gate assembly (NEB Golden Gate Assembly Kit, New England Biolabs, USA) with BsaI–HF and T4 DNA ligase. For multiplex targeting within a single gene, Gly–tRNA–sgRNA cassettes carrying two 20-bp target sequences were similarly PCR-amplified and inserted under the AtU6 promoter.

### Gene-editing plasmid construction system based on CBE and ABE

In this study, gene editing was performed using both ABE and CBE systems: the ABE system comprised the engineered adenine deaminase TadA-8e fused to Cas9 nickase (nCas9–D10A), while the CBE system incorporated the cytosine deaminase variant evoAPOBEC1 fused to nCas9–D10A and a uracil glycosylase inhibitor (UGI). Homologs of Arabidopsis constitutive promoter–related proteins (*AtUBQ10*, *AtACT2*, and *AtTUB4*) were identified in papaya by NCBI BLASTP (E-value <1 × 10^−10^), and 1000 bp upstream of their transcriptional start sites were extracted as candidate promoters, designated *CpUBI*, *CpACT*, and *CpTUB*. Seven promoters (*2 × CaMV 35S*, *CpACT*, *CpTUB*, *CpUBI*, *RpS5A*, *AtUBQ10*, and *CmYLCV*) were amplified using KOD-Plus-Neo DNA Polymerase (Toyobo, KOD-401S) [42]. Backbone vectors carrying deaminases were digested with XmaI/NotI, and promoter fragments were inserted into pIB360RB-A or pIB360RB-C using the ClonExpress II One-Step Cloning Kit (Vazyme, China) [42]. A 23-bp target sequence including the PAM was selected using CRISPR-P2.0 0 [41] and cloned into the vectors via Golden Gate assembly with BsaI–HF and T4 DNA ligase from the NEB Golden Gate Assembly Kit (New England Biolabs, USA). Ligation products were transformed into *Escherichia coli* DH5α and plated on LB agar containing 50 μg/ml kanamycin; positive monoclonal clones were confirmed by Sanger sequencing (TsingKe, China) [42]. Plasmids from verified clones were introduced into *A. rhizogenes* K599 by the freeze–thaw method [43], and positive colonies were selected on TY medium containing kanamycin (50 mg/L) and streptomycin (50 mg/L).

### CDB delivery system and amplicon sequencing

*A. rhizogenes* strain K599 was used to induce hairy roots in papaya. Positive clones were cultured in 1 ml TY liquid medium (50 mg/L kanamycin, 50 mg/L streptomycin) at 28°C (200 rpm, dark) for 18 to 24 h and then streaked onto TY agar, where they were incubated overnight under the same conditions. Healthy papaya explants—including cotyledons, true leaves, and stem segments—were prepared: cotyledons were detached from the stem; true leaves were excised from the basal half of the blade; stem segments were trimmed to remove excess leaves and buds, leaving a tender apical bud. The apical 1 cm of each stem segment was cut at a 45° angle. The cut explants were dipped into the *A. rhizogenes* culture on TY agar and then placed in breathable culture boxes containing 50 g of vermiculite and 100 ml of sterile water. Boxes were sealed and incubated in a greenhouse at 28°C 16 h light/8 h dark photoperiod for 30 days, allowing transgenic hairy roots to develop. Genomic DNA was extracted from the transgenic hairy roots and leaf tissues using the CTAB method [44], and the target site flanking regions were PCR-amplified and sequenced using the Hi-TOM platform to determine editing efficiency [45]. Transgenic roots were identified by their red pigmentation conferred by a RUBY reporter gene. The rooting rate (percentage of infected leaves that produced roots) and the transgenic rate (percentage of infected leaves bearing transgenic roots) were calculated. All primers used for PCR amplification prior to Hi-TOM sequencing are listed in Supplementary Tables S3 and S10.

### Transformation of papaya embryogenic calli

We used plasmid BGK012 (Biogle #BGK012), which carries an *Arabidopsis thaliana* U6 promoter and a codon-optimized human SpCas9, as the backbone for assembling our CRISPR/Cas9 construct (with a hygromycin marker) [46–48]. The plasmid was introduced into *A. tumefaciens* strain GV3101—a common strain for plant transformation. *A. tumefaciens* GV3101 was grown overnight in LB medium (50 mg/L kanamycin, 50 mg/L rifampicin) at 28°C and 200 rpm (in the dark). The cells were harvested by centrifugation, washed twice with sterile half-strength MS (1/2 MS) medium, and resuspended in 1/2 MS to an OD_600_ of about 0.8. Healthy papaya embryogenic calli were then immersed in this Agrobacterium suspension and gently agitated (≈5 min) during a total infection period of ~20 to 25 min (optimized in preliminary tests). After infection, the calli were blotted dry on sterile filter paper and air-dried in a laminar hood for ~30 min to reduce excess bacteria. The infected calli were placed on filter paper over *Ci* co-culture medium (2.215 g/L MS, 70 g/L sucrose, 10 mg/L 2,4-D, pH 6.0) and incubated in the dark for 24 h. They were then transferred to fresh *Ci* medium containing 200 mg/L carbenicillin and 200 mg/L cefotaxim dual-antibiotic selection for 7 days in the dark to suppress Agrobacterium overgrowth. Finally, the calli were moved to shoot induction medium *MTH* (MS basal salts with 0.2 mg/L 6-benzylaminopurine, 0.2 mg/L naphthaleneacetic acid, 50 mg/L hygromycin B, and 100 mg/L timentin) under light. After roughly 3 months on this selective medium, hygromycin-resistant yellow-green shoots began to emerge.

## Supplementary Material

Web_Material_uhag049

## Data Availability

All data generated or analyzed during this study are included in this manuscript and its supplementary information files.
